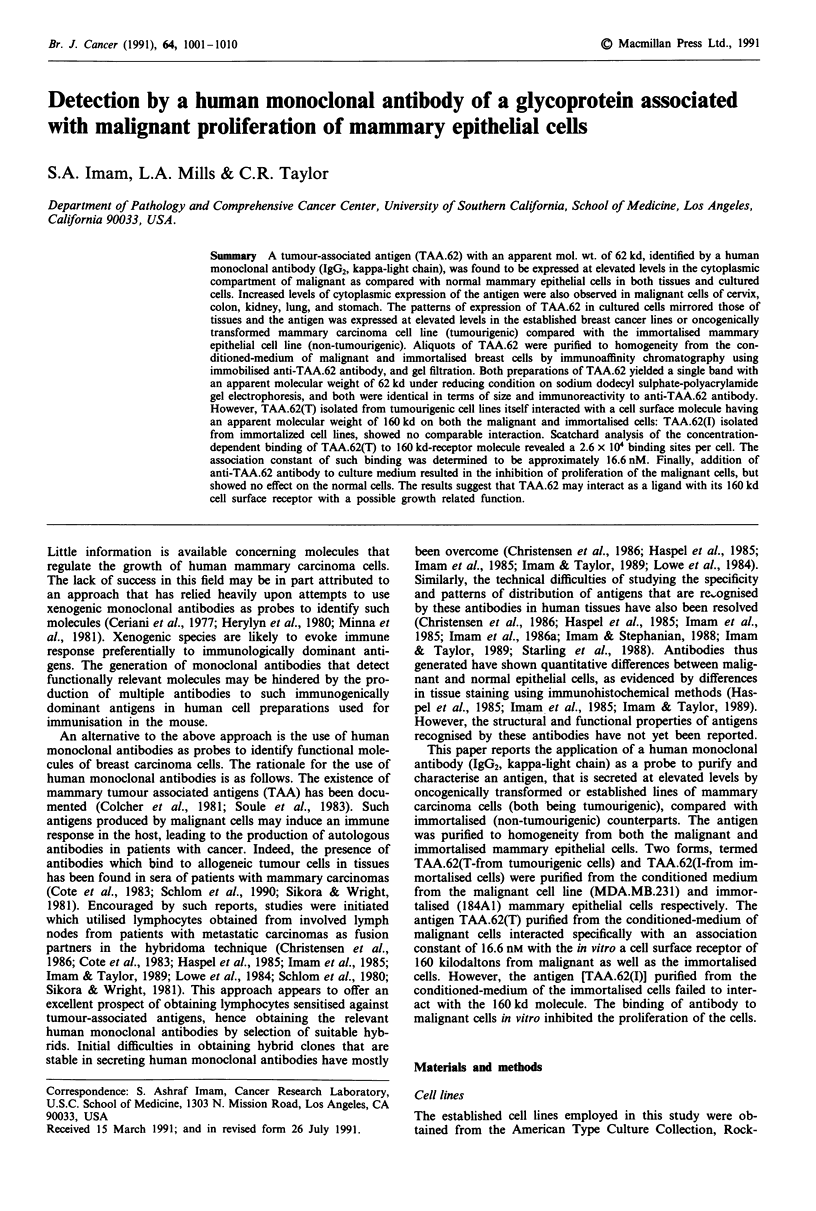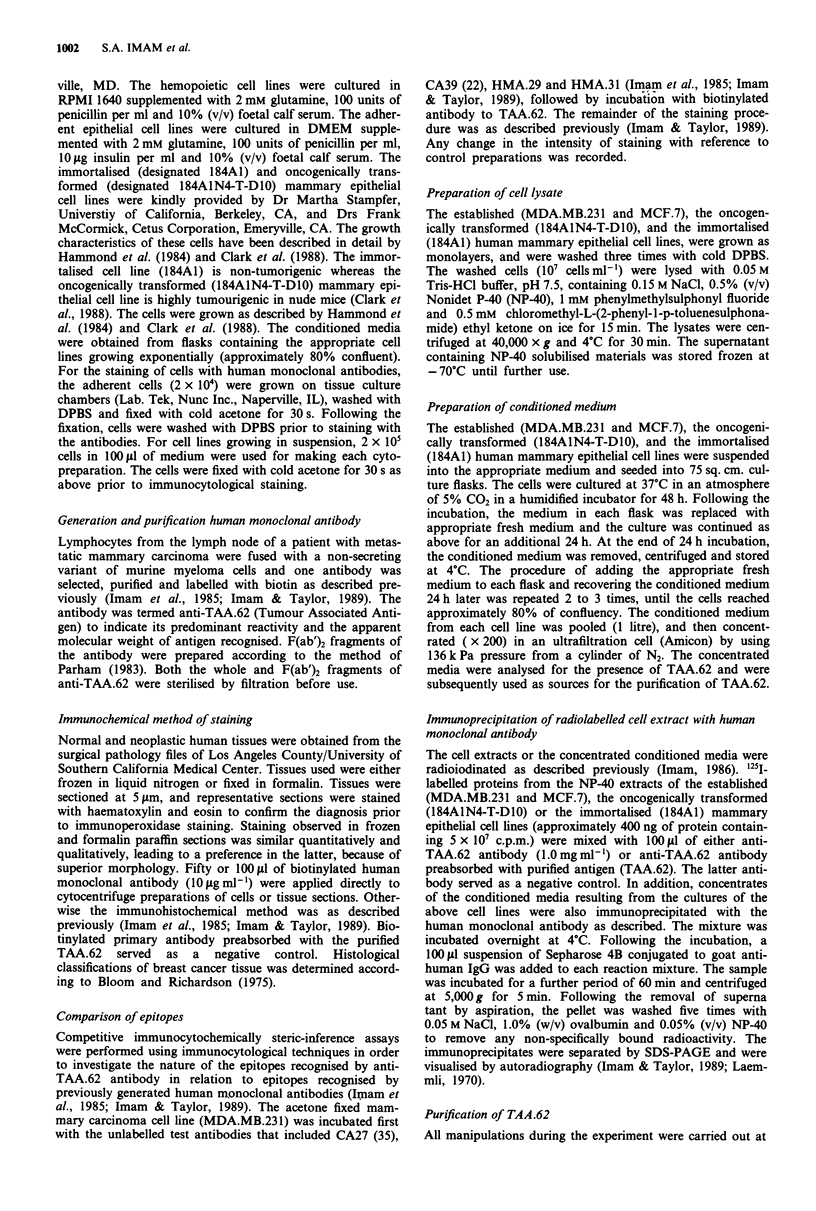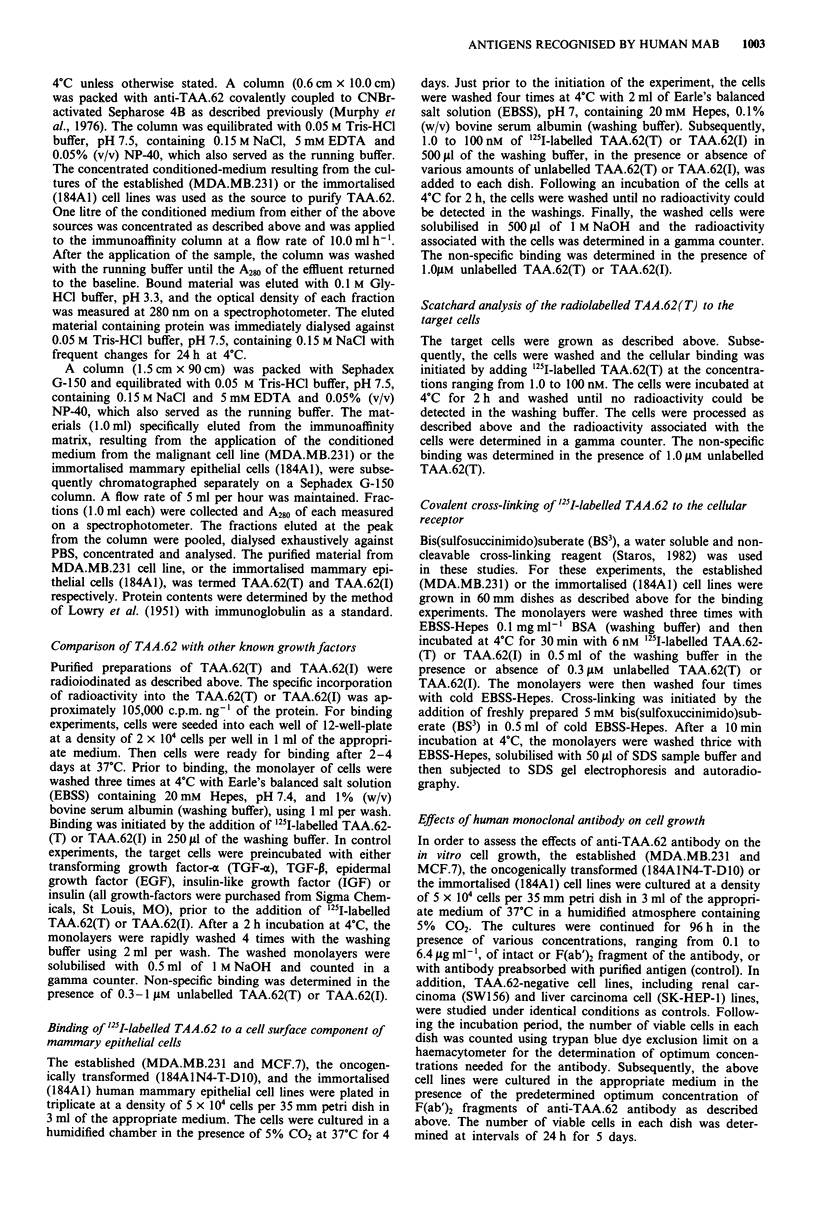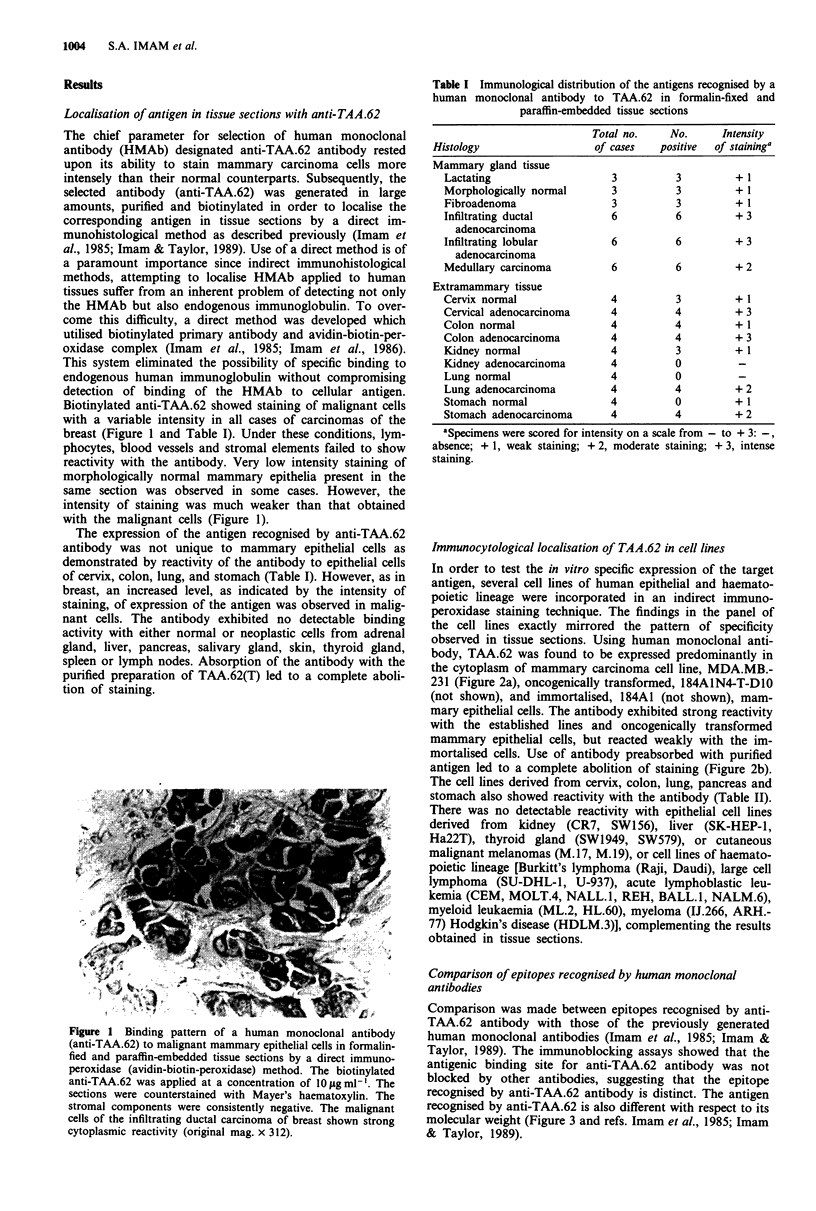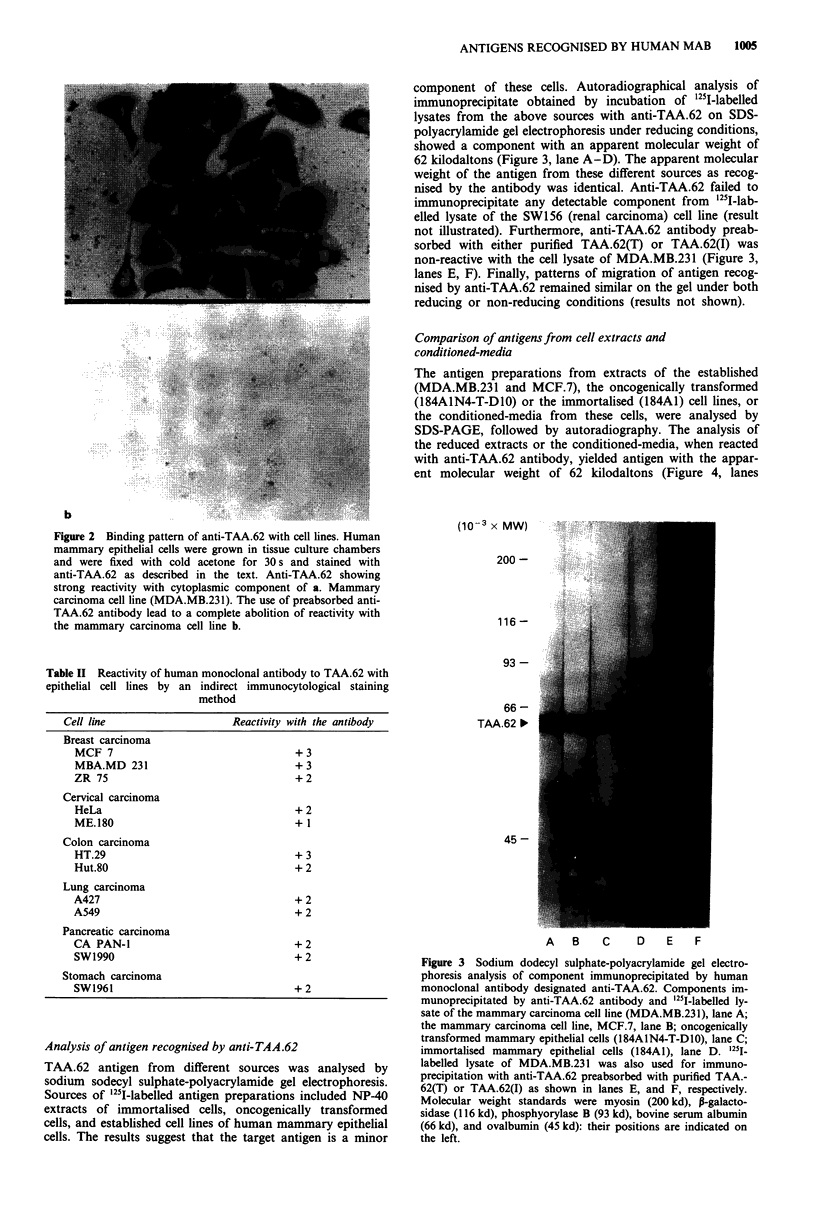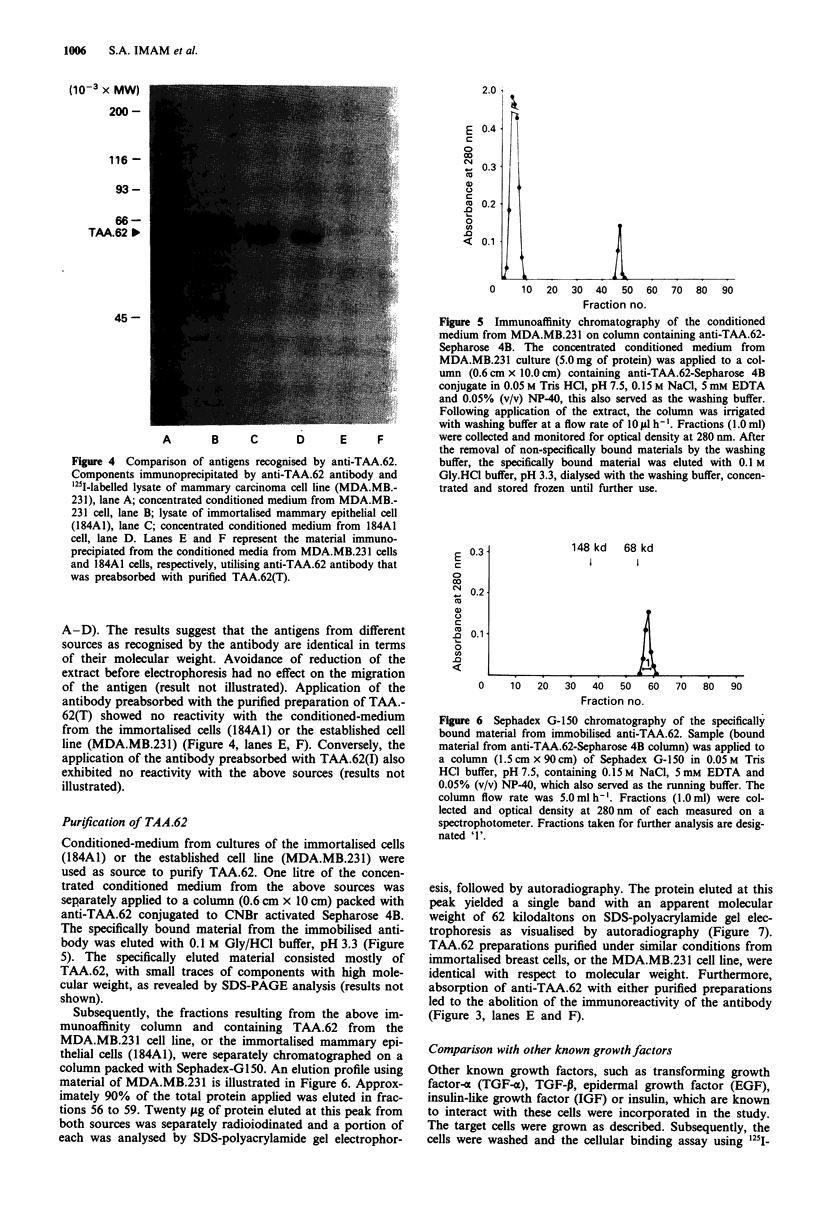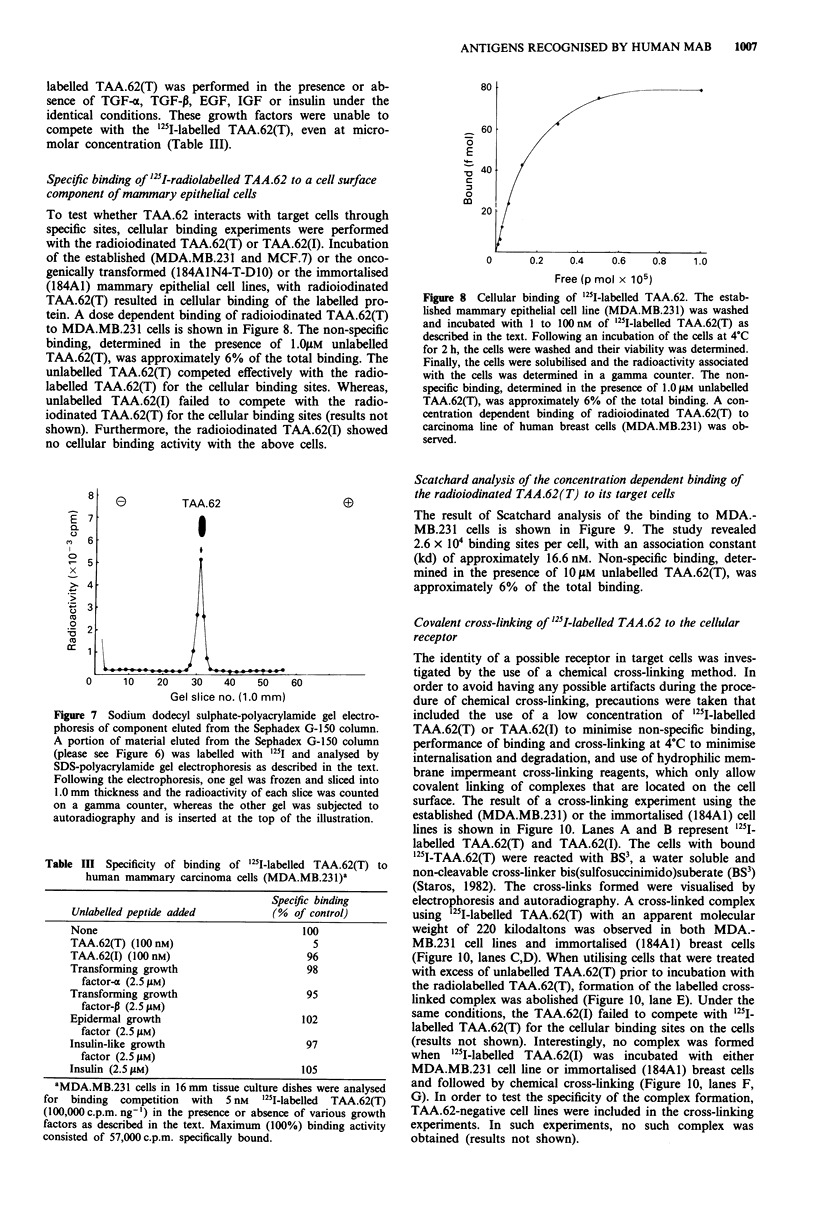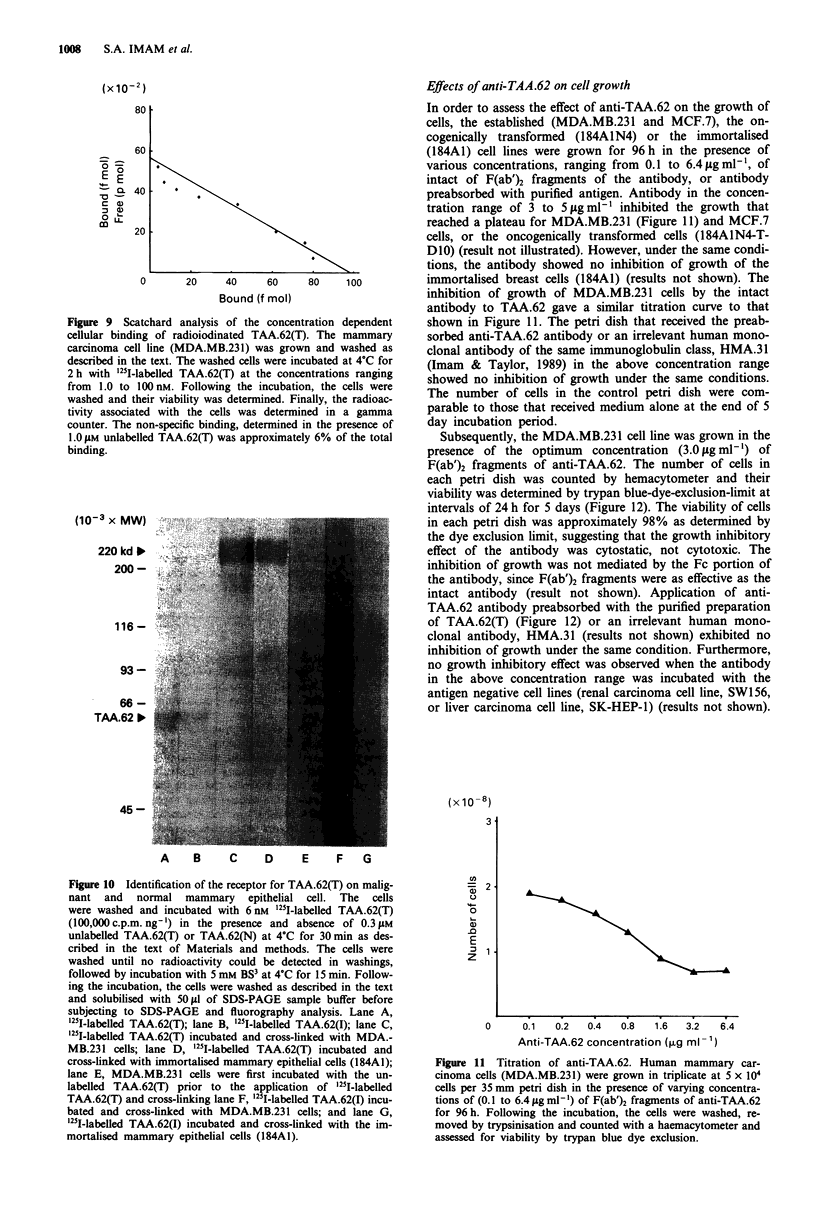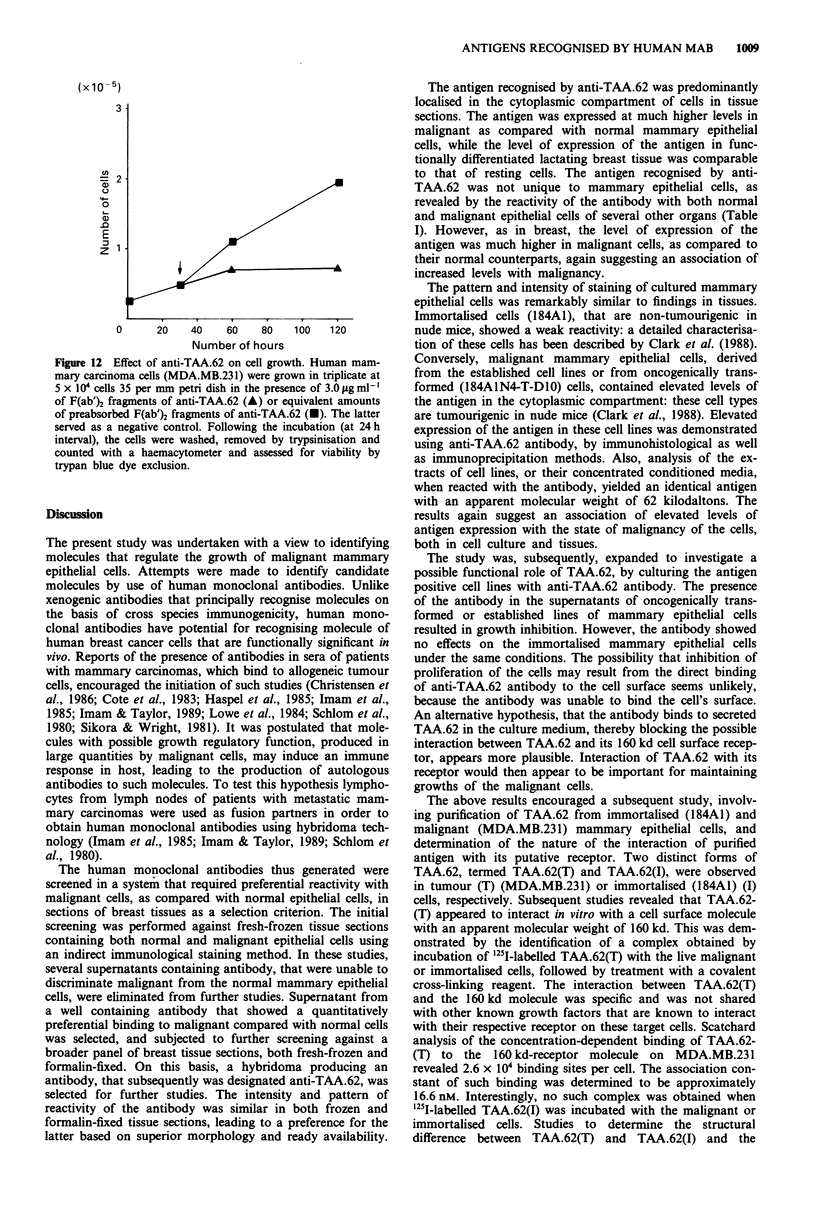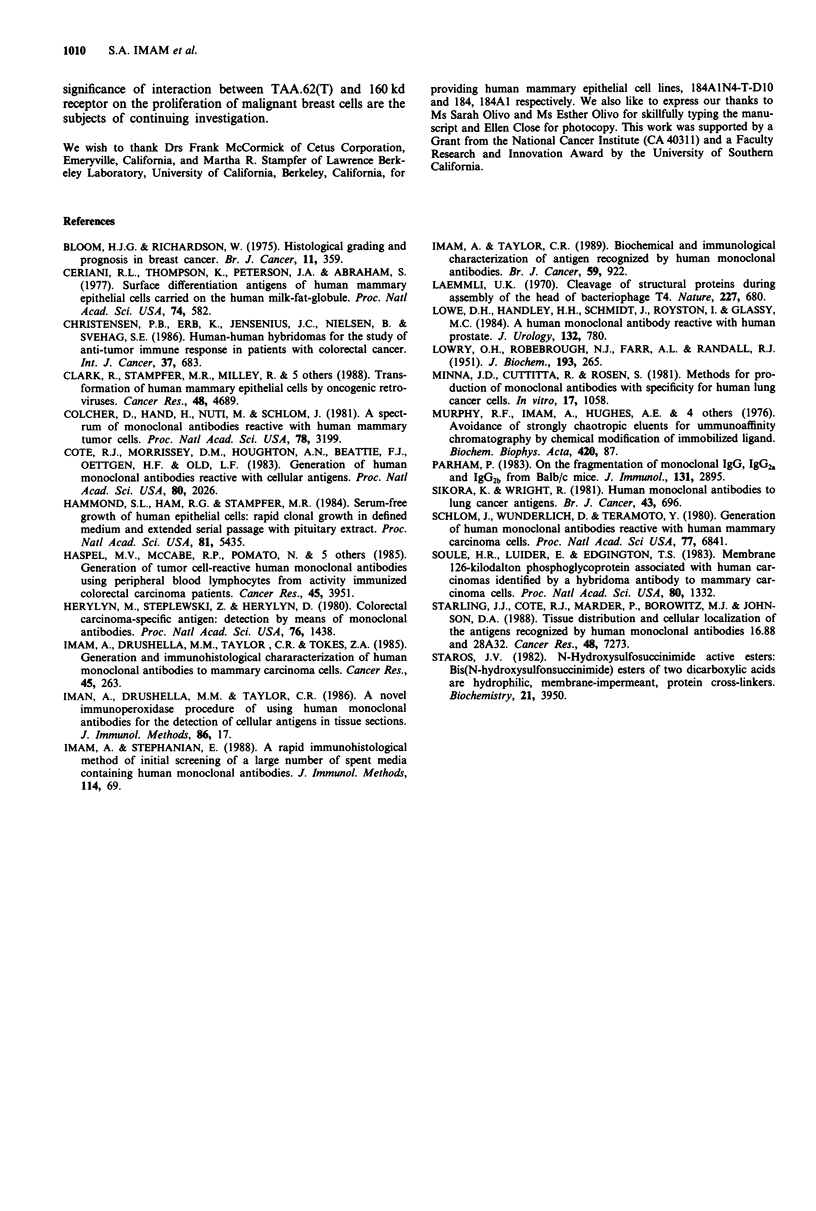# Detection by a human monoclonal antibody of a glycoprotein associated with malignant proliferation of mammary epithelial cells.

**DOI:** 10.1038/bjc.1991.455

**Published:** 1991-12

**Authors:** S. A. Imam, L. A. Mills, C. R. Taylor

**Affiliations:** Department of Pathology, University of Southern California, School of Medicine, Los Angeles 90033.

## Abstract

**Images:**


*Br. J. Cancer* (1991), **64**, 1001–1010 © Macmillan Press Ltd., 1991

### Detection by a human monoclonal antibody of a glycoprotein associated with malignant proliferation of mammary epithelial cells

S.A. Imam, L.A. Mills & C.R. Taylor

*Department of Pathology and Comprehensive Cancer Center, University of Southern California, School of Medicine, Los Angeles, California 90033, USA.*

**Summary** A tumour-associated antigen (TAA.62) with an apparent mol. wt. of 62 kd, identified by a human monoclonal antibody ($IgG_2$, kappa-light chain), was found to be expressed at elevated levels in the cytoplasmic compartment of malignant as compared with normal mammary epithelial cells in both tissues and cultured cells. Increased levels of cytoplasmic expression of the antigen were also observed in malignant cells of cervix, colon, kidney, lung, and stomach. The patterns of expression of TAA.62 in cultured cells mirrored those of tissues and the antigen was expressed at elevated levels in the established breast cancer lines or oncogenically transformed mammary carcinoma cell line (tumourigenic) compared with the immortalised mammary epithelial cell line (non-tumourigenic). Aliquots of TAA.62 were purified to homogeneity from the conditioned-medium of malignant and immortalised breast cells by immunoaffinity chromatography using immobilised anti-TAA.62 antibody, and gel filtration. Both preparations of TAA.62 yielded a single band with an apparent molecular weight of 62 kd under reducing condition on sodium dodecyl sulphate-polyacrylamide gel electrophoresis, and both were identical in terms of size and immunoreactivity to anti-TAA.62 antibody. However, TAA.62(T) isolated from tumourigenic cell lines itself interacted with a cell surface molecule having an apparent molecular weight of 160 kd on both the malignant and immortalised cells: TAA.62(I) isolated from immortalized cell lines, showed no comparable interaction. Scatchard analysis of the concentration-dependent binding of TAA.62(T) to 160 kd-receptor molecule revealed a $2.6 \times 10^4$ binding sites per cell. The association constant of such binding was determined to be approximately 16.6 nM. Finally, addition of anti-TAA.62 antibody to culture medium resulted in the inhibition of proliferation of the malignant cells, but showed no effect on the normal cells. The results suggest that TAA.62 may interact as a ligand with its 160 kd cell surface receptor with a possible growth related function.

Little information is available concerning molecules that regulate the growth of human mammary carcinoma cells. The lack of success in this field may be in part attributed to an approach that has relied heavily upon attempts to use xenogenic monoclonal antibodies as probes to identify such molecules (Ceriani *et al.*, 1977; Herylyn *et al.*, 1980; Minna *et al.*, 1981). Xenogenic species are likely to evoke immune response preferentially to immunologically dominant antigens. The generation of monoclonal antibodies that detect functionally relevant molecules may be hindered by the production of multiple antibodies to such immunogenically dominant antigens in human cell preparations used for immunisation in the mouse.

An alternative to the above approach is the use of human monoclonal antibodies as probes to identify functional molecules of breast carcinoma cells. The rationale for the use of human monoclonal antibodies is as follows. The existence of mammary tumour associated antigens (TAA) has been documented (Colcher *et al.*, 1981; Soule *et al.*, 1983). Such antigens produced by malignant cells may induce an immune response in the host, leading to the production of autologous antibodies in patients with cancer. Indeed, the presence of antibodies which bind to allogeneic tumour cells in tissues has been found in sera of patients with mammary carcinomas (Cote *et al.*, 1983; Schlom *et al.*, 1990; Sikora & Wright, 1981). Encouraged by such reports, studies were initiated which utilised lymphocytes obtained from involved lymph nodes from patients with metastatic carcinomas as fusion partners in the hybridoma technique (Christensen *et al.*, 1986; Cote *et al.*, 1983; Haspel *et al.*, 1985; Imam *et al.*, 1985; Imam & Taylor, 1989; Lowe *et al.*, 1984; Schlom *et al.*, 1980; Sikora & Wright, 1981). This approach appears to offer an excellent prospect of obtaining lymphocytes sensitised against tumour-associated antigens, hence obtaining the relevant human monoclonal antibodies by selection of suitable hybrids. Initial difficulties in obtaining hybrid clones that are stable in secreting human monoclonal antibodies have mostly been overcome (Christensen *et al.*, 1986; Haspel *et al.*, 1985; Imam *et al.*, 1985; Imam & Taylor, 1989; Lowe *et al.*, 1984). Similarly, the technical difficulties of studying the specificity and patterns of distribution of antigens that are recognised by these antibodies in human tissues have also been resolved (Christensen *et al.*, 1986; Haspel *et al.*, 1985; Imam *et al.*, 1985; Imam *et al.*, 1986a; Imam & Stephanian, 1988; Imam & Taylor, 1989; Starling *et al.*, 1988). Antibodies thus generated have shown quantitative differences between malignant and normal epithelial cells, as evidenced by differences in tissue staining using immunohistochemical methods (Haspel *et al.*, 1985; Imam *et al.*, 1985; Imam & Taylor, 1989). However, the structural and functional properties of antigens recognised by these antibodies have not yet been reported.

This paper reports the application of a human monoclonal antibody ($IgG_2$, kappa-light chain) as a probe to purify and characterise an antigen, that is secreted at elevated levels by oncogenically transformed or established lines of mammary carcinoma cells (both being tumourigenic), compared with immortalised (non-tumourigenic) counterparts. The antigen was purified to homogeneity from both the malignant and immortalised mammary epithelial cells. Two forms, termed TAA.62(T-from tumourigenic cells) and TAA.62(I-from immortalised cells) were purified from the conditioned medium from the malignant cell line (MDA.MB.231) and immortalised (184A1) mammary epithelial cells respectively. The antigen TAA.62(T) purified from the conditioned-medium of malignant cells interacted specifically with an association constant of 16.6 nM with the *in vitro* a cell surface receptor of 160 kilodaltons from malignant as well as the immortalised cells. However, the antigen [TAA.62(I)] purified from the conditioned-medium of the immortalised cells failed to interact with the 160 kd molecule. The binding of antibody to malignant cells *in vitro* inhibited the proliferation of the cells.

### Materials and methods

### *Cell lines*

The established cell lines employed in this study were obtained from the American Type Culture Collection, Rock-

Correspondence: S. Ashraf Imam, Cancer Research Laboratory, U.S.C. School of Medicine, 1303 N. Mission Road, Los Angeles, CA 90033, USA
Received 15 March 1991; and in revised form 26 July 1991.

ville, MD. The hemopoietic cell lines were cultured in RPMI 1640 supplemented with 2 mM glutamine, 100 units of penicillin per ml and 10% (v/v) foetal calf serum. The adherent epithelial cell lines were cultured in DMEM supplemented with 2 mM glutamine, 100 units of penicillin per ml, 10 μg insulin per ml and 10% (v/v) foetal calf serum. The immortalised (designated 184A1) and oncogenically transformed (designated 184A1N4-T-D10) mammary epithelial cell lines were kindly provided by Dr Martha Stampfer, Universtiy of California, Berkeley, CA, and Drs Frank McCormick, Cetus Corporation, Emeryville, CA. The growth characteristics of these cells have been described in detail by Hammond et al. (1984) and Clark et al. (1988). The immortalised cell line (184A1) is non-tumorigenic whereas the oncogenically transformed (184A1N4-T-D10) mammary epithelial cell line is highly tumourigenic in nude mice (Clark et al., 1988). The cells were grown as described by Hammond et al. (1984) and Clark et al. (1988). The conditioned media were obtained from flasks containing the appropriate cell lines growing exponentially (approximately 80% confluent). For the staining of cells with human monoclonal antibodies, the adherent cells ($2 \times 10^4$) were grown on tissue culture chambers (Lab. Tek, Nunc Inc., Naperville, IL), washed with DPBS and fixed with cold acetone for 30 s. Following the fixation, cells were washed with DPBS prior to staining with the antibodies. For cell lines growing in suspension, $2 \times 10^5$ cells in 100 μl of medium were used for making each cytopreparation. The cells were fixed with cold acetone for 30 s as above prior to immunocytological staining.

*Generation and purification human monoclonal antibody*

Lymphocytes from the lymph node of a patient with metastatic mammary carcinoma were fused with a non-secreting variant of murine myeloma cells and one antibody was selected, purified and labelled with biotin as described previously (Imam et al., 1985; Imam & Taylor, 1989). The antibody was termed anti-TAA.62 (Tumour Associated Antigen) to indicate its predominant reactivity and the apparent molecular weight of antigen recognised. $F(ab')_2$ fragments of the antibody were prepared according to the method of Parham (1983). Both the whole and $F(ab')_2$ fragments of anti-TAA.62 were sterilised by filtration before use.

*Immunochemical method of staining*

Normal and neoplastic human tissues were obtained from the surgical pathology files of Los Angeles County/University of Southern California Medical Center. Tissues used were either frozen in liquid nitrogen or fixed in formalin. Tissues were sectioned at 5 μm, and representative sections were stained with haematoxylin and eosin to confirm the diagnosis prior to immunoperoxidase staining. Staining observed in frozen and formalin paraffin sections was similar quantitatively and qualitatively, leading to a preference in the latter, because of superior morphology. Fifty or 100 μl of biotinylated human monoclonal antibody (10 μg $ml^{-1}$) were applied directly to cytocentrifuge preparations of cells or tissue sections. Otherwise the immunohistochemical method was as described previously (Imam et al., 1985; Imam & Taylor, 1989). Biotinylated primary antibody preabsorbed with the purified TAA.62 served as a negative control. Histological classifications of breast cancer tissue was determined according to Bloom and Richardson (1975).

*Comparison of epitopes*

Competitive immunocytochemically steric-inference assays were performed using immunocytological techniques in order to investigate the nature of the epitopes recognised by anti-TAA.62 antibody in relation to epitopes recognised by previously generated human monoclonal antibodies (Imam et al., 1985; Imam & Taylor, 1989). The acetone fixed mammary carcinoma cell line (MDA.MB.231) was incubated first with the unlabelled test antibodies that included CA27 (35), CA39 (22), HMA.29 and HMA.31 (Imam et al., 1985; Imam & Taylor, 1989), followed by incubation with biotinylated antibody to TAA.62. The remainder of the staining procedure was as described previously (Imam & Taylor, 1989). Any change in the intensity of staining with reference to control preparations was recorded.

*Preparation of cell lysate*

The established (MDA.MB.231 and MCF.7), the oncogenically transformed (184A1N4-T-D10), and the immortalised (184A1) human mammary epithelial cell lines, were grown as monolayers, and were washed three times with cold DPBS. The washed cells ($10^7$ cells $ml^{-1}$) were lysed with 0.05 M Tris-HCl buffer, pH 7.5, containing 0.15 M NaCl, 0.5% (v/v) Nonidet P-40 (NP-40), 1 mM phenylmethylsulphonyl fluoride and 0.5 mM chloromethyl-L-(2-phenyl-1-p-toluenesulphonamide) ethyl ketone on ice for 15 min. The lysates were centrifuged at 40,000 × *g* and 4°C for 30 min. The supernatant containing NP-40 solubilised materials was stored frozen at −70°C until further use.

*Preparation of conditioned medium*

The established (MDA.MB.231 and MCF.7), the oncogenically transformed (184A1N4-T-D10), and the immortalised (184A1) human mammary epithelial cell lines were suspended into the appropriate medium and seeded into 75 sq. cm. culture flasks. The cells were cultured at 37°C in an atmosphere of 5% $CO_2$ in a humidified incubator for 48 h. Following the incubation, the medium in each flask was replaced with appropriate fresh medium and the culture was continued as above for an additional 24 h. At the end of 24 h incubation, the conditioned medium was removed, centrifuged and stored at 4°C. The procedure of adding the appropriate fresh medium to each flask and recovering the conditioned medium 24 h later was repeated 2 to 3 times, until the cells reached approximately 80% of confluency. The conditioned medium from each cell line was pooled (1 litre), and then concentrated ( × 200) in an ultrafiltration cell (Amicon) by using 136 k Pa pressure from a cylinder of $N_2$. The concentrated media were analysed for the presence of TAA.62 and were subsequently used as sources for the purification of TAA.62.

*Immunoprecipitation of radiolabelled cell extract with human monoclonal antibody*

The cell extracts or the concentrated conditioned media were radioiodinated as described previously (Imam, 1986). $^{125}I$-labelled proteins from the NP-40 extracts of the established (MDA.MB.231 and MCF.7), the oncogenically transformed (184A1N4-T-D10) or the immortalised (184A1) mammary epithelial cell lines (approximately 400 ng of protein containing $5 \times 10^7$ c.p.m.) were mixed with 100 μl of either anti-TAA.62 antibody (1.0 mg $ml^{-1}$) or anti-TAA.62 antibody preabsorbed with purified antigen (TAA.62). The latter antibody served as a negative control. In addition, concentrates of the conditioned media resulting from the cultures of the above cell lines were also immunoprecipitated with the human monoclonal antibody as described. The mixture was incubated overnight at 4°C. Following the incubation, a 100 μl suspension of Sepharose 4B conjugated to goat anti-human IgG was added to each reaction mixture. The sample was incubated for a further period of 60 min and centrifuged at 5,000 *g* for 5 min. Following the removal of superna tant by aspiration, the pellet was washed five times with 0.05 M NaCl, 1.0% (w/v) ovalbumin and 0.05% (v/v) NP-40 to remove any non-specifically bound radioactivity. The immunoprecipitates were separated by SDS-PAGE and were visualised by autoradiography (Imam & Taylor, 1989; Laemmli, 1970).

*Purification of TAA.62*

All manipulations during the experiment were carried out at

4°C unless otherwise stated. A column (0.6 cm × 10.0 cm) was packed with anti-TAA.62 covalently coupled to CNBr-activated Sepharose 4B as described previously (Murphy *et al.*, 1976). The column was equilibrated with 0.05 M Tris-HCl buffer, pH 7.5, containing 0.15 M NaCl, 5 mM EDTA and 0.05% (v/v) NP-40, which also served as the running buffer. The concentrated conditioned-medium resulting from the cultures of the established (MDA.MB.231) or the immortalised (184A1) cell lines was used as the source to purify TAA.62. One litre of the conditioned medium from either of the above sources was concentrated as described above and was applied to the immunoaffinity column at a flow rate of 10.0 ml $h^{-1}$. After the application of the sample, the column was washed with the running buffer until the $A_{280}$ of the effluent returned to the baseline. Bound material was eluted with 0.1 M Gly-HCl buffer, pH 3.3, and the optical density of each fraction was measured at 280 nm on a spectrophotometer. The eluted material containing protein was immediately dialysed against 0.05 M Tris-HCl buffer, pH 7.5, containing 0.15 M NaCl with frequent changes for 24 h at 4°C.

A column (1.5 cm × 90 cm) was packed with Sephadex G-150 and equilibrated with 0.05 M Tris-HCl buffer, pH 7.5, containing 0.15 M NaCl and 5 mM EDTA and 0.05% (v/v) NP-40, which also served as the running buffer. The materials (1.0 ml) specifically eluted from the immunoaffinity matrix, resulting from the application of the conditioned medium from the malignant cell line (MDA.MB.231) or the immortalised mammary epithelial cells (184A1), were subsequently chromatographed separately on a Sephadex G-150 column. A flow rate of 5 ml per hour was maintained. Fractions (1.0 ml each) were collected and $A_{280}$ of each measured on a spectrophotometer. The fractions eluted at the peak from the column were pooled, dialysed exhaustively against PBS, concentrated and analysed. The purified material from MDA.MB.231 cell line, or the immortalised mammary epithelial cells (184A1), was termed TAA.62(T) and TAA.62(I) respectively. Protein contents were determined by the method of Lowry *et al.* (1951) with immunoglobulin as a standard.

### *Comparison of TAA.62 with other known growth factors*

Purified preparations of TAA.62(T) and TAA.62(I) were radioiodinated as described above. The specific incorporation of radioactivity into the TAA.62(T) or TAA.62(I) was approximately 105,000 c.p.m. $ng^{-1}$ of the protein. For binding experiments, cells were seeded into each well of 12-well-plate at a density of $2 \times 10^4$ cells per well in 1 ml of the appropriate medium. Then cells were ready for binding after 2–4 days at 37°C. Prior to binding, the monolayer of cells were washed three times at 4°C with Earle's balanced salt solution (EBSS) containing 20 mM Hepes, pH 7.4, and 1% (w/v) bovine serum albumin (washing buffer), using 1 ml per wash. Binding was initiated by the addition of $^{125}$I-labelled TAA.62-(T) or TAA.62(I) in 250 µl of the washing buffer. In control experiments, the target cells were preincubated with either transforming growth factor-α (TGF-α), TGF-β, epidermal growth factor (EGF), insulin-like growth factor (IGF) or insulin (all growth-factors were purchased from Sigma Chemicals, St Louis, MO), prior to the addition of $^{125}$I-labelled TAA.62(T) or TAA.62(I). After a 2 h incubation at 4°C, the monolayers were rapidly washed 4 times with the washing buffer using 2 ml per wash. The washed monolayers were solubilised with 0.5 ml of 1 M NaOH and counted in a gamma counter. Non-specific binding was determined in the presence of 0.3–1 µM unlabelled TAA.62(T) or TAA.62(I).

### *Binding of $^{125}$I-labelled TAA.62 to a cell surface component of mammary epithelial cells*

The established (MDA.MB.231 and MCF.7), the oncogenically transformed (184A1N4-T-D10), and the immortalised (184A1) human mammary epithelial cell lines were plated in triplicate at a density of $5 \times 10^4$ cells per 35 mm petri dish in 3 ml of the appropriate medium. The cells were cultured in a humidified chamber in the presence of 5% $CO_2$ at 37°C for 4 days. Just prior to the initiation of the experiment, the cells were washed four times at 4°C with 2 ml of Earle's balanced salt solution (EBSS), pH 7, containing 20 mM Hepes, 0.1% (w/v) bovine serum albumin (washing buffer). Subsequently, 1.0 to 100 nM of $^{125}$I-labelled TAA.62(T) or TAA.62(I) in 500 µl of the washing buffer, in the presence or absence of various amounts of unlabelled TAA.62(T) or TAA.62(I), was added to each dish. Following an incubation of the cells at 4°C for 2 h, the cells were washed until no radioactivity could be detected in the washings. Finally, the washed cells were solubilised in 500 µl of 1 M NaOH and the radioactivity associated with the cells was determined in a gamma counter. The non-specific binding was determined in the presence of 1.0µM unlabelled TAA.62(T) or TAA.62(I).

### *Scatchard analysis of the radiolabelled TAA.62(T) to the target cells*

The target cells were grown as described above. Subsequently, the cells were washed and the cellular binding was initiated by adding $^{125}$I-labelled TAA.62(T) at the concentrations ranging from 1.0 to 100 nM. The cells were incubated at 4°C for 2 h and washed until no radioactivity could be detected in the washing buffer. The cells were processed as described above and the radioactivity associated with the cells were determined in a gamma counter. The non-specific binding was determined in the presence of 1.0 µM unlabelled TAA.62(T).

### *Covalent cross-linking of $^{125}$I-labelled TAA.62 to the cellular receptor*

Bis(sulfosuccinimido)suberate ($BS^3$), a water soluble and non-cleavable cross-linking reagent (Staros, 1982) was used in these studies. For these experiments, the established (MDA.MB.231) or the immortalised (184A1) cell lines were grown in 60 mm dishes as described above for the binding experiments. The monolayers were washed three times with EBSS-Hepes 0.1 mg $ml^{-1}$ BSA (washing buffer) and then incubated at 4°C for 30 min with 6 nM $^{125}$I-labelled TAA.62-(T) or TAA.62(I) in 0.5 ml of the washing buffer in the presence or absence of 0.3 µM unlabelled TAA.62(T) or TAA.62(I). The monolayers were then washed four times with cold EBSS-Hepes. Cross-linking was initiated by the addition of freshly prepared 5 mM bis(sulfoxuccinimido)suberate ($BS^3$) in 0.5 ml of cold EBSS-Hepes. After a 10 min incubation at 4°C, the monolayers were washed thrice with EBSS-Hepes, solubilised with 50 µl of SDS sample buffer and then subjected to SDS gel electrophoresis and autoradiography.

### *Effects of human monoclonal antibody on cell growth*

In order to assess the effects of anti-TAA.62 antibody on the *in vitro* cell growth, the established (MDA.MB.231 and MCF.7), the oncogenically transformed (184A1N4-T-D10) or the immortalised (184A1) cell lines were cultured at a density of $5 \times 10^4$ cells per 35 mm petri dish in 3 ml of the appropriate medium of 37°C in a humidified atmosphere containing 5% $CO_2$. The cultures were continued for 96 h in the presence of various concentrations, ranging from 0.1 to 6.4 µg $ml^{-1}$, of intact or $F(ab')_2$ fragment of the antibody, or with antibody preabsorbed with purified antigen (control). In addition, TAA.62-negative cell lines, including renal carcinoma (SW156) and liver carcinoma cell (SK-HEP-1) lines, were studied under identical conditions as controls. Following the incubation period, the number of viable cells in each dish was counted using trypan blue dye exclusion limit on a haemacytometer for the determination of optimum concentrations needed for the antibody. Subsequently, the above cell lines were cultured in the appropriate medium in the presence of the predetermined optimum concentration of $F(ab')_2$ fragments of anti-TAA.62 antibody as described above. The number of viable cells in each dish was determined at intervals of 24 h for 5 days.

### Results

### *Localisation of antigen in tissue sections with anti-TAA.62*

The chief parameter for selection of human monoclonal antibody (HMAb) designated anti-TAA.62 antibody rested upon its ability to stain mammary carcinoma cells more intensely than their normal counterparts. Subsequently, the selected antibody (anti-TAA.62) was generated in large amounts, purified and biotinylated in order to localise the corresponding antigen in tissue sections by a direct immunohistological method as described previously (Imam *et al.*, 1985; Imam & Taylor, 1989). Use of a direct method is of a paramount importance since indirect immunohistological methods, attempting to localise HMAb applied to human tissues suffer from an inherent problem of detecting not only the HMAb but also endogenous immunoglobulin. To overcome this difficulty, a direct method was developed which utilised biotinylated primary antibody and avidin-biotin-peroxidase complex (Imam *et al.*, 1985; Imam *et al.*, 1986). This system eliminated the possibility of specific binding to endogenous human immunoglobulin without compromising detection of binding of the HMAb to cellular antigen. Biotinylated anti-TAA.62 showed staining of malignant cells with a variable intensity in all cases of carcinomas of the breast (Figure 1 and Table I). Under these conditions, lymphocytes, blood vessels and stromal elements failed to show reactivity with the antibody. Very low intensity staining of morphologically normal mammary epithelia present in the same section was observed in some cases. However, the intensity of staining was much weaker than that obtained with the malignant cells (Figure 1).

The expression of the antigen recognised by anti-TAA.62 antibody was not unique to mammary epithelial cells as demonstrated by reactivity of the antibody to epithelial cells of cervix, colon, lung, and stomach (Table I). However, as in breast, an increased level, as indicated by the intensity of staining, of expression of the antigen was observed in malignant cells. The antibody exhibited no detectable binding activity with either normal or neoplastic cells from adrenal gland, liver, pancreas, salivary gland, skin, thyroid gland, spleen or lymph nodes. Absorption of the antibody with the purified preparation of TAA.62(T) led to a complete abolition of staining.

**Figure 1** Binding pattern of a human monoclonal antibody (anti-TAA.62) to malignant mammary epithelial cells in formalin-fied and paraffin-embedded tissue sections by a direct immunoperoxidase (avidin-biotin-peroxidase) method. The biotinylated anti-TAA.62 was applied at a concentration of $10\,\mu g\,ml^{-1}$. The sections were counterstained with Mayer's haematoxylin. The stromal components were consistently negative. The malignant cells of the infiltrating ductal carcinoma of breast shown strong cytoplasmic reactivity (original mag. × 312).

**Table I** Immunological distribution of the antigens recognised by a human monoclonal antibody to TAA.62 in formalin-fixed and paraffin-embedded tissue sections

| *Histology* | *Total no. of cases* | *No. positive* | *Intensity of staining*[a] |
|---|---|---|---|
| Mammary gland tissue | | | |
| Lactating | 3 | 3 | +1 |
| Morphologically normal | 3 | 3 | +1 |
| Fibroadenoma | 3 | 3 | +1 |
| Infiltrating ductal adenocarcinoma | 6 | 6 | +3 |
| Infiltrating lobular adenocarcinoma | 6 | 6 | +3 |
| Medullary carcinoma | 6 | 6 | +2 |
| Extramammary tissue | | | |
| Cervix normal | 4 | 3 | +1 |
| Cervical adenocarcinoma | 4 | 4 | +3 |
| Colon normal | 4 | 4 | +1 |
| Colon adenocarcinoma | 4 | 4 | +3 |
| Kidney normal | 4 | 3 | +1 |
| Kidney adenocarcinoma | 4 | 0 | – |
| Lung normal | 4 | 0 | – |
| Lung adenocarcinoma | 4 | 4 | +2 |
| Stomach normal | 4 | 0 | +1 |
| Stomach adenocarcinoma | 4 | 4 | +2 |

[a]Specimens were scored for intensity on a scale from – to +3: –, absence; +1, weak staining; +2, moderate staining; +3, intense staining.

### *Immunocytological localisation of TAA.62 in cell lines*

In order to test the *in vitro* specific expression of the target antigen, several cell lines of human epithelial and haematopoietic lineage were incorporated in an indirect immunoperoxidase staining technique. The findings in the panel of the cell lines exactly mirrored the pattern of specificity observed in tissue sections. Using human monoclonal antibody, TAA.62 was found to be expressed predominantly in the cytoplasm of mammary carcinoma cell line, MDA.MB.-231 (Figure 2a), oncogenically transformed, 184A1N4-T-D10 (not shown), and immortalised, 184A1 (not shown), mammary epithelial cells. The antibody exhibited strong reactivity with the established lines and oncogenically transformed mammary epithelial cells, but reacted weakly with the immortalised cells. Use of antibody preabsorbed with purified antigen led to a complete abolition of staining (Figure 2b). The cell lines derived from cervix, colon, lung, pancreas and stomach also showed reactivity with the antibody (Table II). There was no detectable reactivity with epithelial cell lines derived from kidney (CR7, SW156), liver (SK-HEP-1, Ha22T), thyroid gland (SW1949, SW579), or cutaneous malignant melanomas (M.17, M.19), or cell lines of haematopoietic lineage [Burkitt's lymphoma (Raji, Daudi), large cell lymphoma (SU-DHL-1, U-937), acute lymphoblastic leukemia (CEM, MOLT.4, NALL.1, REH, BALL.1, NALM.6), myeloid leukaemia (ML.2, HL.60), myeloma (IJ.266, ARH.-77) Hodgkin's disease (HDLM.3)], complementing the results obtained in tissue sections.

### *Comparison of epitopes recognised by human monoclonal antibodies*

Comparison was made between epitopes recognised by anti-TAA.62 antibody with those of the previously generated human monoclonal antibodies (Imam *et al.*, 1985; Imam & Taylor, 1989). The immunoblocking assays showed that the antigenic binding site for anti-TAA.62 antibody was not blocked by other antibodies, suggesting that the epitope recognised by anti-TAA.62 antibody is distinct. The antigen recognised by anti-TAA.62 is also different with respect to its molecular weight (Figure 3 and refs. Imam *et al.*, 1985; Imam & Taylor, 1989).

**Figure 2** Binding pattern of anti-TAA.62 with cell lines. Human mammary epithelial cells were grown in tissue culture chambers and were fixed with cold acetone for 30 s and stained with anti-TAA.62 as described in the text. Anti-TAA.62 showing strong reactivity with cytoplasmic component of **a**. Mammary carcinoma cell line (MDA.MB.231). The use of preabsorbed anti-TAA.62 antibody lead to a complete abolition of reactivity with the mammary carcinoma cell line **b**.

**Table II** Reactivity of human monoclonal antibody to TAA.62 with epithelial cell lines by an indirect immunocytological staining method

| *Cell line* | *Reactivity with the antibody* |
|---|---|
| Breast carcinoma | |
| MCF 7 | +3 |
| MBA.MD 231 | +3 |
| ZR 75 | +2 |
| Cervical carcinoma | |
| HeLa | +2 |
| ME.180 | +1 |
| Colon carcinoma | |
| HT.29 | +3 |
| Hut.80 | +2 |
| Lung carcinoma | |
| A427 | +2 |
| A549 | +2 |
| Pancreatic carcinoma | |
| CA PAN-1 | +2 |
| SW1990 | +2 |
| Stomach carcinoma | |
| SW1961 | +2 |

### *Analysis of antigen recognised by anti-TAA.62*

TAA.62 antigen from different sources was analysed by sodium sodecyl sulphate-polyacrylamide gel electrophoresis. Sources of $^{125}$I-labelled antigen preparations included NP-40 extracts of immortalised cells, oncogenically transformed cells, and established cell lines of human mammary epithelial cells. The results suggest that the target antigen is a minor component of these cells. Autoradiographical analysis of immunoprecipitate obtained by incubation of $^{125}$I-labelled lysates from the above sources with anti-TAA.62 on SDS-polyacrylamide gel electrophoresis under reducing conditions, showed a component with an apparent molecular weight of 62 kilodaltons (Figure 3, lane A–D). The apparent molecular weight of the antigen from these different sources as recognised by the antibody was identical. Anti-TAA.62 failed to immunoprecipitate any detectable component from $^{125}$I-labelled lysate of the SW156 (renal carcinoma) cell line (result not illustrated). Furthermore, anti-TAA.62 antibody preabsorbed with either purified TAA.62(T) or TAA.62(I) was non-reactive with the cell lysate of MDA.MB.231 (Figure 3, lanes E, F). Finally, patterns of migration of antigen recognised by anti-TAA.62 remained similar on the gel under both reducing or non-reducing conditions (results not shown).

### *Comparison of antigens from cell extracts and conditioned-media*

The antigen preparations from extracts of the established (MDA.MB.231 and MCF.7), the oncogenically transformed (184A1N4-T-D10) or the immortalised (184A1) cell lines, or the conditioned-media from these cells, were analysed by SDS-PAGE, followed by autoradiography. The analysis of the reduced extracts or the conditioned-media, when reacted with anti-TAA.62 antibody, yielded antigen with the apparent molecular weight of 62 kilodaltons (Figure 4, lanes

**Figure 3** Sodium dodecyl sulphate-polyacrylamide gel electrophoresis analysis of component immunoprecipitated by human monoclonal antibody designated anti-TAA.62. Components immunoprecipitated by anti-TAA.62 antibody and $^{125}$I-labelled lysate of the mammary carcinoma cell line (MDA.MB.231), lane A; the mammary carcinoma cell line, MCF.7, lane B; oncogenically transformed mammary epithelial cells (184A1N4-T-D10), lane C; immortalised mammary epithelial cells (184A1), lane D. $^{125}$I-labelled lysate of MDA.MB.231 was also used for immunoprecipitation with anti-TAA.62 preabsorbed with purified TAA.-62(T) or TAA.62(I) as shown in lanes E, and F, respectively. Molecular weight standards were myosin (200 kd), β-galactosidase (116 kd), phosphyorylase B (93 kd), bovine serum albumin (66 kd), and ovalbumin (45 kd): their positions are indicated on the left.

**Figure 4** Comparison of antigens recognised by anti-TAA.62. Components immunoprecipitated by anti-TAA.62 antibody and $^{125}$I-labelled lysate of mammary carcinoma cell line (MDA.MB.-231), lane A; concentrated conditioned medium from MDA.MB.-231 cell, lane B; lysate of immortalised mammary epithelial cell (184A1), lane C; concentrated conditioned medium from 184A1 cell, lane D. Lanes E and F represent the material immuno-precipiated from the conditioned media from MDA.MB.231 cells and 184A1 cells, respectively, utilising anti-TAA.62 antibody that was preabsorbed with purified TAA.62(T).

A–D). The results suggest that the antigens from different sources as recognised by the antibody are identical in terms of their molecular weight. Avoidance of reduction of the extract before electrophoresis had no effect on the migration of the antigen (result not illustrated). Application of the antibody preabsorbed with the purified preparation of TAA.-62(T) showed no reactivity with the conditioned-medium from the immortalised cells (184A1) or the established cell line (MDA.MB.231) (Figure 4, lanes E, F). Conversely, the application of the antibody preabsorbed with TAA.62(I) also exhibited no reactivity with the above sources (results not illustrated).

### *Purification of TAA.62*

Conditioned-medium from cultures of the immortalised cells (184A1) or the established cell line (MDA.MB.231) were used as source to purify TAA.62. One litre of the concentrated conditioned medium from the above sources was separately applied to a column (0.6 cm × 10 cm) packed with anti-TAA.62 conjugated to CNBr activated Sepharose 4B. The specifically bound material from the immobilised antibody was eluted with 0.1 M Gly/HCl buffer, pH 3.3 (Figure 5). The specifically eluted material consisted mostly of TAA.62, with small traces of components with high molecular weight, as revealed by SDS-PAGE analysis (results not shown).

Subsequently, the fractions resulting from the above immunoaffinity column and containing TAA.62 from the MDA.MB.231 cell line, or the immortalised mammary epithelial cells (184A1), were separately chromatographed on a column packed with Sephadex-G150. An elution profile using material of MDA.MB.231 is illustrated in Figure 6. Approximately 90% of the total protein applied was eluted in fractions 56 to 59. Twenty μg of protein eluted at this peak from both sources was separately radioiodinated and a portion of each was analysed by SDS-polyacrylamide gel electrophoresis, followed by autoradiography. The protein eluted at this peak yielded a single band with an apparent molecular weight of 62 kilodaltons on SDS-polyacrylamide gel electrophoresis as visualised by autoradiography (Figure 7). TAA.62 preparations purified under similar conditions from immortalised breast cells, or the MDA.MB.231 cell line, were identical with respect to molecular weight. Furthermore, absorption of anti-TAA.62 with either purified preparations led to the abolition of the immunoreactivity of the antibody (Figure 3, lanes E and F).

**Figure 5** Immunoaffinity chromatography of the conditioned medium from MDA.MB.231 on column containing anti-TAA.62-Sepharose 4B. The concentrated conditioned medium from MDA.MB.231 culture (5.0 mg of protein) was applied to a column (0.6 cm × 10.0 cm) containing anti-TAA.62-Sepharose 4B conjugate in 0.05 M Tris HCl, pH 7.5, 0.15 M NaCl, 5 mM EDTA and 0.05% (v/v) NP-40, this also served as the washing buffer. Following application of the extract, the column was irrigated with washing buffer at a flow rate of 10 μl h$^{-1}$. Fractions (1.0 ml) were collected and monitored for optical density at 280 nm. After the removal of non-specifically bound materials by the washing buffer, the specifically bound material was eluted with 0.1 M Gly.HCl buffer, pH 3.3, dialysed with the washing buffer, concentrated and stored frozen until further use.

**Figure 6** Sephadex G-150 chromatography of the specifically bound material from immobilised anti-TAA.62. Sample (bound material from anti-TAA.62-Sepharose 4B column) was applied to a column (1.5 cm × 90 cm) of Sephadex G-150 in 0.05 M Tris HCl buffer, pH 7.5, containing 0.15 M NaCl, 5 mM EDTA and 0.05% (v/v) NP-40, which also served as the running buffer. The column flow rate was 5.0 ml h$^{-1}$. Fractions (1.0 ml) were collected and optical density at 280 nm of each measured on a spectrophotometer. Fractions taken for further analysis are designated '1'.

### *Comparison with other known growth factors*

Other known growth factors, such as transforming growth factor-α (TGF-α), TGF-β, epidermal growth factor (EGF), insulin-like growth factor (IGF) or insulin, which are known to interact with these cells were incorporated in the study. The target cells were grown as described. Subsequently, the cells were washed and the cellular binding assay using $^{125}$I-

labelled TAA.62(T) was performed in the presence or absence of TGF-α, TGF-β, EGF, IGF or insulin under the identical conditions. These growth factors were unable to compete with the $^{125}$I-labelled TAA.62(T), even at micromolar concentration (Table III).

*Specific binding of $^{125}$I-radiolabelled TAA.62 to a cell surface component of mammary epithelial cells*

To test whether TAA.62 interacts with target cells through specific sites, cellular binding experiments were performed with the radioiodinated TAA.62(T) or TAA.62(I). Incubation of the established (MDA.MB.231 and MCF.7) or the oncogenically transformed (184A1N4-T-D10) or the immortalised (184A1) mammary epithelial cell lines, with radioiodinated TAA.62(T) resulted in cellular binding of the labelled protein. A dose dependent binding of radioiodinated TAA.62(T) to MDA.MB.231 cells is shown in Figure 8. The non-specific binding, determined in the presence of 1.0μM unlabelled TAA.62(T), was approximately 6% of the total binding. The unlabelled TAA.62(T) competed effectively with the radiolabelled TAA.62(T) for the cellular binding sites. Whereas, unlabelled TAA.62(I) failed to compete with the radioiodinated TAA.62(T) for the cellular binding sites (results not shown). Furthermore, the radioiodinated TAA.62(I) showed no cellular binding activity with the above cells.

**Figure 7** Sodium dodecyl sulphate-polyacrylamide gel electrophoresis of component eluted from the Sephadex G-150 column. A portion of material eluted from the Sephadex G-150 column (please see Figure 6) was labelled with $^{125}$I and analysed by SDS-polyacrylamide gel electrophoresis as described in the text. Following the electrophoresis, one gel was frozen and sliced into 1.0 mm thickness and the radioactivity of each slice was counted on a gamma counter, whereas the other gel was subjected to autoradiography and is inserted at the top of the illustration.

**Table III** Specificity of binding of $^{125}$I-labelled TAA.62(T) to human mammary carcinoma cells (MDA.MB.231)[a]

| *Unlabelled peptide added* | *Specific binding (% of control)* |
|---|---|
| None | 100 |
| TAA.62(T) (100 nM) | 5 |
| TAA.62(I) (100 nM) | 96 |
| Transforming growth factor-α (2.5 μM) | 98 |
| Transforming growth factor-β (2.5 μM) | 95 |
| Epidermal growth factor (2.5 μM) | 102 |
| Insulin-like growth factor (2.5 μM) | 97 |
| Insulin (2.5 μM) | 105 |

[a]MDA.MB.231 cells in 16 mm tissue culture dishes were analysed for binding competition with 5 nM $^{125}$I-labelled TAA.62(T) (100,000 c.p.m. $ng^{-1}$) in the presence or absence of various growth factors as described in the text. Maximum (100%) binding activity consisted of 57,000 c.p.m. specifically bound.

**Figure 8** Cellular binding of $^{125}$I-labelled TAA.62. The established mammary epithelial cell line (MDA.MB.231) was washed and incubated with 1 to 100 nM of $^{125}$I-labelled TAA.62(T) as described in the text. Following an incubation of the cells at 4°C for 2 h, the cells were washed and their viability was determined. Finally, the cells were solubilised and the radioactivity associated with the cells was determined in a gamma counter. The non-specific binding, determined in the presence of 1.0 μM unlabelled TAA.62(T), was approximately 6% of the total binding. A concentration dependent binding of radioiodinated TAA.62(T) to carcinoma line of human breast cells (MDA.MB.231) was observed.

*Scatchard analysis of the concentration dependent binding of the radioiodinated TAA.62(T) to its target cells*

The result of Scatchard analysis of the binding to MDA.-MB.231 cells is shown in Figure 9. The study revealed $2.6 \times 10^4$ binding sites per cell, with an association constant (kd) of approximately 16.6 nM. Non-specific binding, determined in the presence of 10 μM unlabelled TAA.62(T), was approximately 6% of the total binding.

*Covalent cross-linking of $^{125}$I-labelled TAA.62 to the cellular receptor*

The identity of a possible receptor in target cells was investigated by the use of a chemical cross-linking method. In order to avoid having any possible artifacts during the procedure of chemical cross-linking, precautions were taken that included the use of a low concentration of $^{125}$I-labelled TAA.62(T) or TAA.62(I) to minimise non-specific binding, performance of binding and cross-linking at 4°C to minimise internalisation and degradation, and use of hydrophilic membrane impermeant cross-linking reagents, which only allow covalent linking of complexes that are located on the cell surface. The result of a cross-linking experiment using the established (MDA.MB.231) or the immortalised (184A1) cell lines is shown in Figure 10. Lanes A and B represent $^{125}$I-labelled TAA.62(T) and TAA.62(I). The cells with bound $^{125}$I-TAA.62(T) were reacted with $BS^3$, a water soluble and non-cleavable cross-linker bis(sulfosuccinimido)suberate ($BS^3$) (Staros, 1982). The cross-links formed were visualised by electrophoresis and autoradiography. A cross-linked complex using $^{125}$I-labelled TAA.62(T) with an apparent molecular weight of 220 kilodaltons was observed in both MDA.-MB.231 cell lines and immortalised (184A1) breast cells (Figure 10, lanes C,D). When utilising cells that were treated with excess of unlabelled TAA.62(T) prior to incubation with the radiolabelled TAA.62(T), formation of the labelled cross-linked complex was abolished (Figure 10, lane E). Under the same conditions, the TAA.62(I) failed to compete with $^{125}$I-labelled TAA.62(T) for the cellular binding sites on the cells (results not shown). Interestingly, no complex was formed when $^{125}$I-labelled TAA.62(I) was incubated with either MDA.MB.231 cell line or immortalised (184A1) breast cells and followed by chemical cross-linking (Figure 10, lanes F, G). In order to test the specificity of the complex formation, TAA.62-negative cell lines were included in the cross-linking experiments. In such experiments, no such complex was obtained (results not shown).

**Figure 9** Scatchard analysis of the concentration dependent cellular binding of radioiodinated TAA.62(T). The mammary carcinoma cell line (MDA.MB.231) was grown and washed as described in the text. The washed cells were incubated at 4°C for 2 h with $^{125}$I-labelled TAA.62(T) at the concentrations ranging from 1.0 to 100 nM. Following the incubation, the cells were washed and their viability was determined. Finally, the radioactivity associated with the cells was determined in a gamma counter. The non-specific binding, determined in the presence of 1.0 μM unlabelled TAA.62(T) was approximately 6% of the total binding.

**Figure 10** Identification of the receptor for TAA.62(T) on malignant and normal mammary epithelial cell. The cells were washed and incubated with 6 nM $^{125}$I-labelled TAA.62(T) (100,000 c.p.m. ng$^{-1}$) in the presence and absence of 0.3 μM unlabelled TAA.62(T) or TAA.62(N) at 4°C for 30 min as described in the text of Materials and methods. The cells were washed until no radioactivity could be detected in washings, followed by incubation with 5 mM BS$^3$ at 4°C for 15 min. Following the incubation, the cells were washed as described in the text and solubilised with 50 μl of SDS-PAGE sample buffer before subjecting to SDS-PAGE and fluorography analysis. Lane A, $^{125}$I-labelled TAA.62(T); lane B, $^{125}$I-labelled TAA.62(I); lane C, $^{125}$I-labelled TAA.62(T) incubated and cross-linked with MDA.-MB.231 cells; lane D, $^{125}$I-labelled TAA.62(T) incubated and cross-linked with immortalised mammary epithelial cells (184A1); lane E, MDA.MB.231 cells were first incubated with the unlabelled TAA.62(T) prior to the application of $^{125}$I-labelled TAA.62(T) and cross-linking lane F, $^{125}$I-labelled TAA.62(I) incubated and cross-linked with MDA.MB.231 cells; and lane G, $^{125}$I-labelled TAA.62(I) incubated and cross-linked with the immortalised mammary epithelial cells (184A1).

*Effects of anti-TAA.62 on cell growth*

In order to assess the effect of anti-TAA.62 on the growth of cells, the established (MDA.MB.231 and MCF.7), the oncogenically transformed (184A1N4) or the immortalised (184A1) cell lines were grown for 96 h in the presence of various concentrations, ranging from 0.1 to 6.4 μg ml$^{-1}$, of intact of F(ab')$_2$ fragments of the antibody, or antibody preabsorbed with purified antigen. Antibody in the concentration range of 3 to 5 μg ml$^{-1}$ inhibited the growth that reached a plateau for MDA.MB.231 (Figure 11) and MCF.7 cells, or the oncogenically transformed cells (184A1N4-T-D10) (result not illustrated). However, under the same conditions, the antibody showed no inhibition of growth of the immortalised breast cells (184A1) (results not shown). The inhibition of growth of MDA.MB.231 cells by the intact antibody to TAA.62 gave a similar titration curve to that shown in Figure 11. The petri dish that received the preabsorbed anti-TAA.62 antibody or an irrelevant human monoclonal antibody of the same immunoglobulin class, HMA.31 (Imam & Taylor, 1989) in the above concentration range showed no inhibition of growth under the same conditions. The number of cells in the control petri dish were comparable to those that received medium alone at the end of 5 day incubation period.

Subsequently, the MDA.MB.231 cell line was grown in the presence of the optimum concentration (3.0 μg ml$^{-1}$) of F(ab')$_2$ fragments of anti-TAA.62. The number of cells in each petri dish was counted by hemacytometer and their viability was determined by trypan blue-dye-exclusion-limit at intervals of 24 h for 5 days (Figure 12). The viability of cells in each petri dish was approximately 98% as determined by the dye exclusion limit, suggesting that the growth inhibitory effect of the antibody was cytostatic, not cytotoxic. The inhibition of growth was not mediated by the Fc portion of the antibody, since F(ab')$_2$ fragments were as effective as the intact antibody (result not shown). Application of anti-TAA.62 antibody preabsorbed with the purified preparation of TAA.62(T) (Figure 12) or an irrelevant human monoclonal antibody, HMA.31 (results not shown) exhibited no inhibition of growth under the same condition. Furthermore, no growth inhibitory effect was observed when the antibody in the above concentration range was incubated with the antigen negative cell lines (renal carcinoma cell line, SW156, or liver carcinoma cell line, SK-HEP-1) (results not shown).

**Figure 11** Titration of anti-TAA.62. Human mammary carcinoma cells (MDA.MB.231) were grown in triplicate at $5 \times 10^4$ cells per 35 mm petri dish in the presence of varying concentrations of (0.1 to 6.4 μg ml$^{-1}$) of F(ab')$_2$ fragments of anti-TAA.62 for 96 h. Following the incubation, the cells were washed, removed by trypsinisation and counted with a haemacytometer and assessed for viability by trypan blue dye exclusion.

**Figure 12** Effect of anti-TAA.62 on cell growth. Human mammary carcinoma cells (MDA.MB.231) were grown in triplicate at $5 \times 10^4$ cells 35 per mm petri dish in the presence of 3.0 $\mu g\, ml^{-1}$ of $F(ab')_2$ fragments of anti-TAA.62 (▲) or equivalent amounts of preabsorbed $F(ab')_2$ fragments of anti-TAA.62 (■). The latter served as a negative control. Following the incubation (at 24 h interval), the cells were washed, removed by trypsinisation and counted with a haemacytometer and assessed for viability by trypan blue dye exclusion.

### Discussion

The present study was undertaken with a view to identifying molecules that regulate the growth of malignant mammary epithelial cells. Attempts were made to identify candidate molecules by use of human monoclonal antibodies. Unlike xenogenic antibodies that principally recognise molecules on the basis of cross species immunogenicity, human monoclonal antibodies have potential for recognising molecule of human breast cancer cells that are functionally significant *in vivo*. Reports of the presence of antibodies in sera of patients with mammary carcinomas, which bind to allogeneic tumour cells, encouraged the initiation of such studies (Christensen *et al.*, 1986; Cote *et al.*, 1983; Haspel *et al.*, 1985; Imam *et al.*, 1985; Imam & Taylor, 1989; Lowe *et al.*, 1984; Schlom *et al.*, 1980; Sikora & Wright, 1981). It was postulated that molecules with possible growth regulatory function, produced in large quantities by malignant cells, may induce an immune response in host, leading to the production of autologous antibodies to such molecules. To test this hypothesis lymphocytes from lymph nodes of patients with metastatic mammary carcinomas were used as fusion partners in order to obtain human monoclonal antibodies using hybridoma technology (Imam *et al.*, 1985; Imam & Taylor, 1989; Schlom *et al.*, 1980).

The human monoclonal antibodies thus generated were screened in a system that required preferential reactivity with malignant cells, as compared with normal epithelial cells, in sections of breast tissues as a selection criterion. The initial screening was performed against fresh-frozen tissue sections containing both normal and malignant epithelial cells using an indirect immunological staining method. In these studies, several supernatants containing antibody, that were unable to discriminate malignant from the normal mammary epithelial cells, were eliminated from further studies. Supernatant from a well containing antibody that showed a quantitatively preferential binding to malignant compared with normal cells was selected, and subjected to further screening against a broader panel of breast tissue sections, both fresh-frozen and formalin-fixed. On this basis, a hybridoma producing an antibody, that subsequently was designated anti-TAA.62, was selected for further studies. The intensity and pattern of reactivity of the antibody was similar in both frozen and formalin-fixed tissue sections, leading to a preference for the latter based on superior morphology and ready availability.

The antigen recognised by anti-TAA.62 was predominantly localised in the cytoplasmic compartment of cells in tissue sections. The antigen was expressed at much higher levels in malignant as compared with normal mammary epithelial cells, while the level of expression of the antigen in functionally differentiated lactating breast tissue was comparable to that of resting cells. The antigen recognised by anti-TAA.62 was not unique to mammary epithelial cells, as revealed by the reactivity of the antibody with both normal and malignant epithelial cells of several other organs (Table I). However, as in breast, the level of expression of the antigen was much higher in malignant cells, as compared to their normal counterparts, again suggesting an association of increased levels with malignancy.

The pattern and intensity of staining of cultured mammary epithelial cells was remarkably similar to findings in tissues. Immortalised cells (184A1), that are non-tumourigenic in nude mice, showed a weak reactivity: a detailed characterisation of these cells has been described by Clark *et al.* (1988). Conversely, malignant mammary epithelial cells, derived from the established cell lines or from oncogenically transformed (184A1N4-T-D10) cells, contained elevated levels of the antigen in the cytoplasmic compartment: these cell types are tumourigenic in nude mice (Clark *et al.*, 1988). Elevated expression of the antigen in these cell lines was demonstrated using anti-TAA.62 antibody, by immunohistological as well as immunoprecipitation methods. Also, analysis of the extracts of cell lines, or their concentrated conditioned media, when reacted with the antibody, yielded an identical antigen with an apparent molecular weight of 62 kilodaltons. The results again suggest an association of elevated levels of antigen expression with the state of malignancy of the cells, both in cell culture and tissues.

The study was, subsequently, expanded to investigate a possible functional role of TAA.62, by culturing the antigen positive cell lines with anti-TAA.62 antibody. The presence of the antibody in the supernatants of oncogenically transformed or established lines of mammary epithelial cells resulted in growth inhibition. However, the antibody showed no effects on the immortalised mammary epithelial cells under the same conditions. The possibility that inhibition of proliferation of the cells may result from the direct binding of anti-TAA.62 antibody to the cell surface seems unlikely, because the antibody was unable to bind the cell's surface. An alternative hypothesis, that the antibody binds to secreted TAA.62 in the culture medium, thereby blocking the possible interaction between TAA.62 and its 160 kd cell surface receptor, appears more plausible. Interaction of TAA.62 with its receptor would then appear to be important for maintaining growths of the malignant cells.

The above results encouraged a subsequent study, involving purification of TAA.62 from immortalised (184A1) and malignant (MDA.MB.231) mammary epithelial cells, and determination of the nature of the interaction of purified antigen with its putative receptor. Two distinct forms of TAA.62, termed TAA.62(T) and TAA.62(I), were observed in tumour (T) (MDA.MB.231) or immortalised (184A1) (I) cells, respectively. Subsequent studies revealed that TAA.62-(T) appeared to interact *in vitro* with a cell surface molecule with an apparent molecular weight of 160 kd. This was demonstrated by the identification of a complex obtained by incubation of $^{125}I$-labelled TAA.62(T) with the live malignant or immortalised cells, followed by treatment with a covalent cross-linking reagent. The interaction between TAA.62(T) and the 160 kd molecule was specific and was not shared with other known growth factors that are known to interact with their respective receptor on these target cells. Scatchard analysis of the concentration-dependent binding of TAA.62-(T) to the 160 kd-receptor molecule on MDA.MB.231 revealed $2.6 \times 10^4$ binding sites per cell. The association constant of such binding was determined to be approximately 16.6 nM. Interestingly, no such complex was obtained when $^{125}I$-labelled TAA.62(I) was incubated with the malignant or immortalised cells. Studies to determine the structural difference between TAA.62(T) and TAA.62(I) and the significance of interaction between TAA.62(T) and 160 kd receptor on the proliferation of malignant breast cells are the subjects of continuing investigation.

We wish to thank Drs Frank McCormick of Cetus Corporation, Emeryville, California, and Martha R. Stampfer of Lawrence Berkeley Laboratory, University of California, Berkeley, California, for providing human mammary epithelial cell lines, 184A1N4-T-D10 and 184, 184A1 respectively. We also like to express our thanks to Ms Sarah Olivo and Ms Esther Olivo for skillfully typing the manuscript and Ellen Close for photocopy. This work was supported by a Grant from the National Cancer Institute (CA 40311) and a Faculty Research and Innovation Award by the University of Southern California.

### References

BLOOM, H.J.G. & RICHARDSON, W. (1975). Histological grading and prognosis in breast cancer. *Br. J. Cancer*, **11**, 359.

CERIANI, R.L., THOMPSON, K., PETERSON, J.A. & ABRAHAM, S. (1977). Surface differentiation antigens of human mammary epithelial cells carried on the human milk-fat-globule. *Proc. Natl Acad. Sci. USA*, **74**, 582.

CHRISTENSEN, P.B., ERB, K., JENSENIUS, J.C., NIELSEN, B. & SVEHAG, S.E. (1986). Human-human hybridomas for the study of anti-tumor immune response in patients with colorectal cancer. *Int. J. Cancer*, **37**, 683.

CLARK, R., STAMPFER, M.R., MILLEY, R. & 5 others (1988). Transformation of human mammary epithelial cells by oncogenic retroviruses. *Cancer Res.*, **48**, 4689.

COLCHER, D., HAND, H., NUTI, M. & SCHLOM, J. (1981). A spectrum of monoclonal antibodies reactive with human mammary tumor cells. *Proc. Natl Acad. Sci. USA*, **78**, 3199.

COTE, R.J., MORRISSEY, D.M., HOUGHTON, A.N., BEATTIE, F.J., OETTGEN, H.F. & OLD, L.F. (1983). Generation of human monoclonal antibodies reactive with cellular antigens. *Proc. Natl Acad. Sci. USA*, **80**, 2026.

HAMMOND, S.L., HAM, R.G. & STAMPFER, M.R. (1984). Serum-free growth of human epithelial cells: rapid clonal growth in defined medium and extended serial passage with pituitary extract. *Proc. Natl Acad. Sci. USA*, **81**, 5435.

HASPEL, M.V., MCCABE, R.P., POMATO, N. & 5 others (1985). Generation of tumor cell-reactive human monoclonal antibodies using peripheral blood lymphocytes from activity immunized colorectal carcinoma patients. *Cancer Res.*, **45**, 3951.

HERYLYN, M., STEPLEWSKI, Z. & HERYLYN, D. (1980). Colorectal carcinoma-specific antigen: detection by means of monoclonal antibodies. *Proc. Natl Acad. Sci. USA*, **76**, 1438.

IMAM, A., DRUSHELLA, M.M., TAYLOR , C.R. & TOKES, Z.A. (1985). Generation and immunohistological chararacterization of human monoclonal antibodies to mammary carcinoma cells. *Cancer Res.*, **45**, 263.

IMAN, A., DRUSHELLA, M.M. & TAYLOR, C.R. (1986). A novel immunoperoxidase procedure of using human monoclonal antibodies for the detection of cellular antigens in tissue sections. *J. Immunol. Methods*, **86**, 17.

IMAM, A. & STEPHANIAN, E. (1988). A rapid immunohistological method of initial screening of a large number of spent media containing human monoclonal antibodies. *J. Immunol. Methods*, **114**, 69.

IMAM, A. & TAYLOR, C.R. (1989). Biochemical and immunological characterization of antigen recognized by human monoclonal antibodies. *Br. J. Cancer*, **59**, 922.

LAEMMLI, U.K. (1970). Cleavage of structural proteins during assembly of the head of bacteriophage T4. *Nature*, **227**, 680.

LOWE, D.H., HANDLEY, H.H., SCHMIDT, J., ROYSTON, I. & GLASSY, M.C. (1984). A human monoclonal antibody reactive with human prostate. *J. Urology*, **132**, 780.

LOWRY, O.H., ROBEBROUGH, N.J., FARR, A.L. & RANDALL, R.J. (1951). *J. Biochem.*, **193**, 265.

MINNA, J.D., CUTTITTA, R. & ROSEN, S. (1981). Methods for production of monoclonal antibodies with specificity for human lung cancer cells. *In vitro*, **17**, 1058.

MURPHY, R.F., IMAM, A., HUGHES, A.E. & 4 others (1976). Avoidance of strongly chaotropic eluents for ummunoaffinity chromatography by chemical modification of immobilized ligand. *Biochem. Biophys. Acta*, **420**, 87.

PARHAM, P. (1983). On the fragmentation of monoclonal IgG, $IgG_{2a}$ and $IgG_{2b}$ from Balb/c mice. *J. Immunol.*, **131**, 2895.

SIKORA, K. & WRIGHT, R. (1981). Human monoclonal antibodies to lung cancer antigens. *Br. J. Cancer*, **43**, 696.

SCHLOM, J., WUNDERLICH, D. & TERAMOTO, Y. (1980). Generation of human monoclonal antibodies reactive with human mammary carcinoma cells. *Proc. Natl Acad. Sci USA*, **77**, 6841.

SOULE, H.R., LUIDER, E. & EDGINGTON, T.S. (1983). Membrane 126-kilodalton phosphoglycoprotein associated with human carcinomas identified by a hybridoma antibody to mammary carcinoma cells. *Proc. Natl Acad. Sci. USA*, **80**, 1332.

STARLING, J.J., COTE, R.J., MARDER, P., BOROWITZ, M.J. & JOHNSON, D.A. (1988). Tissue distribution and cellular localization of the antigens recognized by human monoclonal antibodies 16.88 and 28A32. *Cancer Res.*, **48**, 7273.

STAROS, J.V. (1982). N-Hydroxysulfosuccinimide active esters: Bis(N-hydroxysulfonsuccinimide) esters of two dicarboxylic acids are hydrophilic, membrane-impermeant, protein cross-linkers. *Biochemistry*, **21**, 3950.